# Electronic cigarette: a grand deception?

**DOI:** 10.1590/1516-3180.2025.1433.15042025

**Published:** 2025-06-06

**Authors:** Jaqueline Ribeiro Scholz, Sara Del Vecchio Ziotti, Paulo Manuel Pêgo Fernandes

**Affiliations:** I Tobacco Treatment Program, Department of Prevention and Rehabilitation, Tobacco Treatment, Instituto do Coração (InCor), Hospital das Clínicas, Faculdade de Medicina, Universidade de São Paulo (USP), São Paulo (SP), Brazil.; II Department of Prevention and Rehabilitation, Tobacco Treatment, Instituto do Coração (InCor), Hospital das Clínicas, Faculdade de Medicina, Universidade de São Paulo (USP), São Paulo (SP), Brazil.; III Faculdade de Medicina, Universidade de São Paulo (USP), São Paulo (SP), Brazil; Full Professor, Department of Cardiopulmonary Diseases, Faculdade de Medicina, Universidade de São Paulo (USP), São Paulo (SP), Brazil; Director, Scientific Department, Associação Paulista de Medicina (APM), São Paulo (SP), Brazil.

Electronic smoking devices (ESDs), commonly referred to as vapes, are gadgets that heat a liquid solution containing nicotine and other substances to produce an aerosol inhaled by the user. The sale, importation, and advertising of ESDs have been prohibited in Brazil since 2009, a ban reaffirmed in 2024 by Collegiate Board Resolution (RDC) No. 855/2024.^1^ The primary reasons for this ban include the absence of scientific evidence supporting their effectiveness in smoking cessation, their high addictive potential, their increasing use among children and adolescents, their associated short- and long-term health risks, and the threat they pose to Brazil’s national tobacco control policy.^2^ Despite this prohibition, ESD consumption reached 2.5% of the Brazilian population in 2020 and stabilized at 2.1% in 2023,^3^ likely influenced by public debates regarding the ratification of the original ban instituted in 2008. The Sociedade Brasileira de Cardiologia (SBC) strongly advocates for the continuation of this prohibition, citing significant risks to individual and public health, environmental harm, and regulatory and economic challenges for the country.^2^ This editorial aims to underscore the key points justifying the ongoing ban on the commercial sale of ESDs in Brazil and to present compelling data on the high addictive potential of these devices, as revealed by a national study.

Instituto do Coração (InCor), in collaboration with the São Paulo State Health Surveillance Agency and the Toxicology Laboratory of Faculdade de Medicina da Universidade de São Paulo (FMUSP), conducted a cross-sectional observational study to characterize ESD users in the State of São Paulo. The study assessed user behavior, risk perception, socioeconomic profile, and usage patterns (article submitted for publication). A total of 417 participants aged 18 years and older were included between March and September 2024. They were recruited at bars, concerts, and events across various cities in São Paulo and reported exclusive use of ESDs. Exclusion criteria included individuals who used conventional tobacco products or non-tobacco substances (e.g., *Cannabis*) or had an exhaled carbon monoxide concentration of 4 ppm or higher, eliminating combustible product users. Saliva samples were collected to measure nicotine and cotinine levels.

Sociodemographic data indicated that most users were young, white, upper-middle-class individuals with a high educational level. Approximately 60% of users had no prior history of conventional smoking, suggesting that ESDs may serve as a gateway to nicotine use rather than a safe alternative for smoking cessation.

Participants who reported no subjective sense of addiction had an average salivary nicotine level (equivalent to plasma concentrations)^4^ of 138 ng/ml. In contrast, those who reported high addiction had an average level of 435 ng/ml, with some individuals reaching as high as 4,800 ng/ml. For comparison, patients undergoing smoking cessation treatment after smoking 20 cigarettes per day for 25 years had an average nicotine level of 396 ng/ml. This indicates that within just one year of ESD use, individuals can achieve nicotine levels comparable to those of long-term smokers.^5^ Notably, some participants exhibited nicotine concentrations up to six times higher than traditional smokers, highlighting the rapid development of dependence. Increased nicotine levels are likely linked to the type of nicotine used—nicotine salts have a pharmacokinetic profile that allows for higher serum concentrations—as well as the delivery system.^6^ ESDs deliver nicotine more intensely than conventional cigarettes, and usage frequency is significantly higher, with ESD users taking up to 773 puffs per day, three to four times more than the average conventional smoker.^7^ More than 60% of users reported unsuccessful attempts to quit on their own, with this figure rising to 80% among those with higher dependence, reinforcing the strong addictive potential of vapes.

Even in countries with more robust legal and regulatory frameworks, such as the United States, Australia, the United Kingdom, New Zealand, and France, legislation regarding ESDs is under revision due to the significant increase in use among children and adolescents.^8^ Moreover, these devices have been enhanced to include larger e-liquid reservoirs, higher nicotine concentrations, and reduced costs, all of which facilitate increased use and addiction.^9^

Scientific evidence indicates that ESDs not only promote nicotine dependence but may also pose greater health risks than conventional tobacco. The high bioavailability of nicotine, combined with frequent use, contributes to a rapid onset of addiction. Increased nicotine exposure, particularly when initiated at a young age, elevates the risk of developing chronic diseases.

Effective public policies, grounded in robust scientific evidence, are urgently needed to curb the growing prevalence of these devices, especially among young users. A critical challenge is to prevent regression in the progress achieved by Brazil’s successful tobacco control policies.
